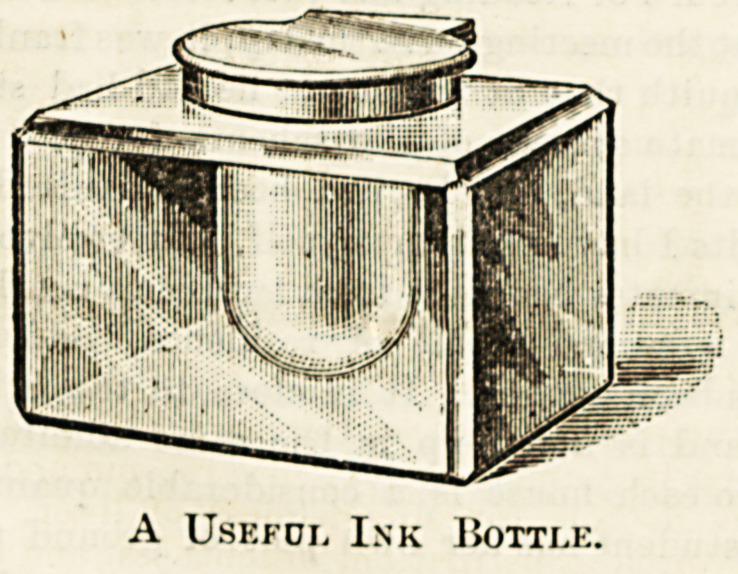# "The Hospital" Nursing Mirror

**Published:** 1899-12-16

**Authors:** 


### "The Hospital" Nursing Mirror.

Being the Nursing Section of "The Hospital."

[Contributions for this Section of "The Hospital" should be addressed to the Editor, The Hospital, 28 & 29, Southampton Street, Strand, London, W.C., and should have the word "Nursing" plainly written in left-hand top corner of the envelope.]

### Notes on News from the Nursing World.

### THE PRINCESS OF WALES'S WAR FUND.

We are now prepared to receive subscriptions of from one to five shillings from nurses to the Princess of Wales's hospital ship, in accordance with the wishes of a very large number of our readers. In no case must the amount exceed five shillings. Subscriptions should be forwarded, in an envelope containing the name and full address of the sender, to the Manager, The Hospital, 28 and 29, Southampton Street, Strand, W.C. The names of the subscribers will appear in the "Mirror" from week to week, and the list will close on Wednesday, January 24th, 1900, when the result will be conveyed to Her Royal Highness. We append the touching Christmas message of the Princess to the wives or widows of our soldiers in South Africa, conveyed through the medium of the *Woman's Weekly*:—

"Sandringham, December 9.

"My heart bleeds for the poor widows and fatherless whose loved ones have met a glorious death fighting for their Queen and country. May God help and comfort them in their saddened Christmas and give them that peace which passeth understanding:—

"Peace, perfect peace, with loved ones far away?
In Jesu's keeping we are safe and they.

"Alexandra, Princess of Wales."

### NURSING THE WOUNDED.

The poor fellows who have been made as comfortable as possible at Herbert Hospital, Woolwich, do not include any of the wounded soldiers. The first arrival of these will take place in the course of a few weeks, and Miss Gray and her staff will then have their hands very full. If good nursing can ensure a quick recovery it will be provided, and good fare also. The present inmates of Herbert Hospital get as much chicken as they can eat, and they are not refused champagne if it seems desirable that it should be given. Every preparation is being made for the reception and nursing of all our temporarily disabled soldiers. In addition to the military hospitals at Netley and Woolwich, an entire ward at Haslar Naval Hospital has been set apart for their accommodation, and 160 beds are being got ready at Devonport Military Hospital. It is only too probable that the members of the Army Nursing Reserve will be kept busy for some time to come.

### THE HOSPITAL SHIPS.

It is satisfactory to learn that after a trial of her machinery at Sheerness, the "Princess of Wales" was able on Monday to resume her voyage to South Africa. The "Maine" will be on view on Monday only, at 11 Warehouse, West India Transport Docks, the nearest station being West India Dock, which is reached from Fenchurch Street. All applications for tickets should be sent to the honorary secretary of the fund, Mrs. Blow, Walsingham House, Piccadilly. On Saturday the staff of the Portland Field Hospital—a very different sort of thing from a hospital ship—which provides 104 beds, and has a personnel of about 50, left Southampton in the "Tantallon Castle." A number of nurses and students assembled at Waterloo to give a good send off to the four nurses. The hospital is to be sent up country from the Cape, probably to De Aar, but it is entirely at the disposal of the authorities.

### THE NURSES OF THE "MAINE" AND THE MATRONS' COUNCIL.

Last week Miss M. E. Hibbard and her colleagues were the guests of the Matrons' Council at the rooms of the Medical Society in Chandos Street. They were received by Miss Isla Stewart, matron of St. Bartholomew's Hospital, and principal of the Matrons' Council, with whom was Mrs. Bedford Fenwick. In addition to the lady nurses of the "Maine," some of the male nurses were present, and Miss Hibbard, in her brief address, specially alluded to the value and efficiency of their work. It was the intention of Miss Hibbard to read a paper on "The Evolution of the Army Nurse in America." But, as she explained, the subject demanded careful and thoughtful handling, and she had not had time to do it justice.

"But," she said, "the most practical demonstration of the evolution of the army nurse in the United States was that five sisters were present, an evolution that was not not dreamt of at the beginning of the American-Spanish war, when the civil nurses of America were first called upon to aid the army in the care of its sick and wounded. Now they stood on another rung of the ladder, and came filled with most earnest desires to help to take care of the soldiers of England, in whom, she assured them, they felt the truest interest. She and her colleagues wished to take that opportunity of thanking their friends in England for the many courtesies extended to them since their arrival in this country. It was the pride and privilege of nurses to go where and whenever they were most needed, their compensation being the knowledge that they had relieved suffering. The greatest honour had been conferred upon her, her colleagues, and their profession by the generous act of Her Majesty in receiving them at Windsor and sending them out with such kind words and appreciation that would ever live in their hearts and make their lives hereafter far more consecrated to their work, in which they hoped to ever bear in mind that love, loyalty, patience, and harmony with each other should be their paramount motive."

Many ladies and gentlemen were subsequently presented to the American nurses. The guests generally expressed their appreciation of Mrs. Hunt's Ladies Orchestra, who played a charming selection of popular music which was particularly acceptable during an interval of fire light. The electric light suddenly went out, and it was some little time before it could be set going again.

### THE PERILS OF NURSING THE BOERS.

According to the *Natal Witness*, the nursing of Boers by English nurses is attended by very serious risks. Our contemporary recites the experiences of Mrs. Weir, one of the Red Cross nurses, who was engaged in ministering to the troops at the battle of Talana Hill. She also tended some of the Boer wounded, and this is how she was treated: Whilst administering beef-tea to a prostrate Dutchman in the hospital she received a severe kick in the back from one of the ambulance attendants. He apparently thought that she intended to poison his charge, and he thus signified his desire that she should discontinue her attentions. The kick was a most brutal one, and caused Mrs. Weir's retirement from active service. Mrs. Weir, who has now nearly recovered from the savage attack, is at present in Maritzburg, and hopes shortly to resume her nursing duties. But we should like to know whether this glaring instance of brutality and ignorance has been punished by the Boer authorities.

### A WOUNDED OFFICER ON THE NURSES IN SOUTH AFRICA.

The following extract from a letter written by an officer of the 1st Manchester, who was wounded at Elands Laagte, appeared in the *Pall Mall Gazette* on Wednesday:—

"When we arrived at Ladysmith I was carried to the head hospital and was dressed by Major David Bruce. He was awfully nice and kind, though frighfully busy, and up to his eyes in blood. The bullet was still in my leg, and he decided to have me put under the X rays next day and see if he could find it. I felt quite certain that the bullet was in my right hip, though it had gone in just below the groin of the right leg, just missing the femoral artery by a hair's breadth. Jolly lucky, wasn't it, that it wasn't a quarter of an inch higher? Yesterday I had a bit of a doing, being lifted from my bed on to a stretcher and carried to the Town Hall, lifted from the stretcher, and placed under the X rays, where I was for about an hour. While there I discovered the bullet in my hip, and as it was not very deep in I was put on the stretcher and carried off to the operating theatre. I had had breakfast, so could not have chloroform, and Major Bruce cut it out. It did hurt a bit, I must confess, but I was jolly glad to have it out. It is a nice solid Mauser rifle bullet, and does not seem to be any the worse for hitting me. I shall have a hole put in it when I go down to Pietermaritzburg, and have the date engraved on it and the name of the action—Elands Laagte—and shall keep it as a record. . . . I am feeling a good deal better now, and really the treatment here is quite wonderful. The nurses in this ward are perfect dears, and nothing is too much trouble for them, though they are most frightfully hardworked. There are twenty-two beds in this ward, and on Sunday and Monday they were all occupied by men who could do next to nothing for themselves. There are only two nurses and two orderlies for the ward, and so you may guess the work is pretty hard."

### THE KILBURN SISTERS AND THE WAR.

A correspondent, whose letter appears elsewhere, personally vouches for the qualifications of one of the Kilburn Sisters to undertake the nursing of the sick and wounded in South Africa. The lady in question, our correspondent states, was trained at the London Hospital, and held appointments as matron and sister in other hospitals before joining the Kilburn Sisterhood. This is conclusive as to the question of the training of that particular member of the sisterhood. But there are hundreds of nurses who have been equally well-trained anxious to go to South Africa, and though we think that more may be needed, if, indeed, they are not needed already, we remain of opinion that independent organisations, not recognising official authority, are not desirable.

### THE QUEEN'S NURSES IN SCOTLAND.

The eleventh annual report of the Scottish branch of the Queen Victoria Jubilee Institute for Nurses, which has just been issued, shows that during the year ending October 31st, 1899, 16 candidates entered the training home for the required month of probation before receiving hospital training, and only one failed; that 34 probationers who had received hospital training entered the home for six months' district training, 8 of whom had received their two years' general hospital training by arrangement with the Institute; and that 33 nurses completed their full training as Queen's nurses in Edinburgh, an increase of 5 on the previous year. In the Higginbotham Home 10 nurses entered for district training during the year, and 2 in the Paisley Home; while 4 received three months' training in the Edinburgh City Hospital, and 3 the same period of training in the Glasgow Maternity Hospital. During the year 10 new branches have been affiliated to the Institute, and have engaged Queen's nurses. There are now no less than 187 Queen's nurses working in Scotland under 103 associations. The Home in Castle Terrace, Edinburgh, has been necessarily enlarged at a cost, including furnishing, of about £600, part of the funds required being obtained by a sale of work, to which the Queen and the Princess Henry of Battenberg contributed.

### THE AMERICAN AUXILIARY SOCIETY OF TRAINED NURSES.

The head-quarters of the Auxiliary Society of Trained Nurses are in an old Spanish mansion in Malate, in the environs of Manila; and a Manila paper, referring to the institution, says: "From this picturesque casa, hidden by flowering shrubs and banana trees, there constantly emanates a wealth of thoughtfulness, solicitude, and loving care for our soldiers, and to it are constantly returned the unstinted gratitude of alleviated humanity." The number of nurses in charge of Miss Henshall, who was head nurse at one of the large military hospitals in New York during the Spanish war, is 27, and the society supplements on lines of its own the work of the Hospital Corps. It has charge of all the wards of one hospital and six wards of another. This means the care of about 600 patients. As the climate is very trying, the nurses will be changed at the end of six months. The work is also arduous, for one of the nurses says that she has not been off for a single day since her arrival, and she laughs at the mere idea of an afternoon nap.

### CHRISTMAS DECORATIONS AT GUY'S HOSPITAL.

In accordance with time-honoured traditions, Guy's Hospital will again be decorated during Christmas week. The practice is to allow the decorations to remain *in situ* for one week only—Christmas Day to New Year's Day. On January 1st every scrap of the decorative material has to be removed from the wards. It does not, therefore, remain on the walls long enough to collect much dust or cause any septic trouble. The work of decorating is undertaken by the students, and the nurses are entirely relieved of any trouble in the matter. Various materials are employed for decoration, though little art muslin is used. Some of the designs are very artistic and elaborate, and for a week before Christmas the medical students are busy in their leisure time preparing the decorations. They have also to clear away the materials on New Year's Day. In the new wards no nails are allowed to be driven in the walls, but there are hooks fixed in the ceilings to which the decorations may be attached. In the old wards nails may be used to fasten the wreaths and festoons, but the authorities consider this a bad system and discourage it, although it has been permitted for years. The students are very ingenious in fixing up a kind of trellis work to which the decorative materials are attached. Everything possible is done to make Christmas week in the hospital bright and happy for the patients, and the authorities regard decorations as an important auxiliary in this aim. The students not only decorate the hospital, but they contribute very liberally to the entertainment of the patients. A good dinner of roast beef and plum pudding is supplied by the hospital on Christmas Day, but the students make a collection to add a number of *et ceteras*, and to provide quite an elaborate tea. Nearly all the wards get turkeys, and as a special privilege the male patients are allowed to smoke. As much as 2s. 6d. per bed is contributed by the medical staff to add to the Christmas good cheer. The students also have a nigger minstrel troupe, which entertains the wards in succession during Christmas week. On these occasions the patients are allowed to sit up an hour longer than usual, but the entertainment must be over and the ward left in quietness by nine o'clock. Christmas week in the hospital is like no other time of the year, and every effort is put forward by both the medical staff and the sisters to add to the enjoyment of the patients. A good deal of clothing for necessitous patients is sent in by ladies' needlework guilds.

### CHRISTMAS AT ST. MARY'S HOSPITAL.

Music will be liberally provided during the Christmas season at St. Mary's Hospital. In every ward where the health of the patients permits, a piano will be placed for a week, beginning on Christmas Eve. On that night festivities will commence with carol singing by the students and nurses in the hall, and the strains will penetrate into the decorated wards. Santa Claus distributes his bounty during the night, and the patients spend the first hours of Christmas Day in examining his gifts. Seasonable cheer in the shape of beef, turkey, and pudding, is served at dinner, and in the afternoon the patients receive their friends to tea. The children have a Christmas tree in the board-room on Boxing Day, when an abundance of toys, a Punch and Judy show, and all sorts of harmless delicacies, are provided for their entertainment. The resident staff gives an evening entertainment to the out-patients early in January, and another to the sisters, nurses, and their friends, at a later date. Great care is taken to prevent disturbing noises reaching the wards containing the graver cases. At the same time, no effort is spared to make Christmas happy and joyous to all. The gifts are partly provided by the St. Mary's Aid Society, of which the founder and president is Mrs. Russell Cooke.

### NURSES AT CANNES.

We have received from the Rev. W. M. Wollaston, chaplain of St. Paul's, Cannes, and Canon of Gibraltar, a letter in which he desires, through our columns, to warn hospital nurses against going to Cannes, or, indeed, the French Riviera generally, in the hope of finding employment. In the course of his communication Canon Wollaston says, "Nurses are absolutely not wanted, and, if they come, they will only find themselves thrown on public charity." He states that there is already an excellent organisation for supplying nurses, to which doctors are bound to apply when a nurse is wanted, before seeking assistance elsewhere, and it is only "very occasionally, when Cannes is very full," that outside nurses are employed. This winter, he adds, it is not likely to be very full. Canon Wollaston intimates that he has written "after consultation with a doctor and with the lady who manages the nurses' establishment."

### THE NURSES' QUARTERS AT BATH INFIRMARY.

As the result of an inspection of the Bath Workhouse Infirmary, Mr. Wethered, the Local Government Board Inspector, reports that there is "the most pressing and important necessity" for providing accommodation for the whole staff in a separate building. Special reference is made by Mr. Wethered to the unsatisfactory accommodation for the night nurses as regards access and escape in case of fire and other reasons. The report, which also deals with the question of the isolation wards, has been discussed by the Board of Guardians, but a final decision has been postponed. We observe, however, that the Chairman remarked that, while it was for the Local Government Board to advise action as to the isolation wards, the accommodation for the nurses was a matter for the Guardians. This is so undoubtedly, and the sooner the Guardians improve the nurses' quarters the more likely they are to avoid frequent vacancies.

### SHORT ITEMS.

The Baroness Emily Halkett is going to South Africa to assist in nursing the sick and wounded. She was trained at St. Bartholomew's Hospital, and afterwards undertook private nursing. — On Thursday last week a large company assembled, at the invitation of the Committee of Management, to view the new Royal London Ophthalmic Hospital in City Road, the guests expressing much pleasure with all that they saw.—A very successful sale of fancy articles for Christmas was held at the Boys' Surgical Home, Banstead, on Wednesday last. The attendance was large, and a substantial addition to the funds of the home was realised.—The third of the series of entertainments during the winter months for the amusement of the patients at the Cancer Hospital, Brompton, was given on Thursday evening last by the Misses Yetts and friends, the various characters being well sustained and much appreciated by the patients and nurses.—The first entertainment after the reopening of the wards of the Royal Orthopædic Hospital took place last week, when the members of Mr. Will Weed's Drawing-room Party kindly gave a concert to the patients and nurses. The programme consisted of solos, duets, trios, and quartettes on the piano, banjo, and mandoline, also numerous humorous songs, nearly all of which were encored by the audience.—A very successful Bal Poudré was given on Tuesday evening at the Galleries of the Royal Society of Painters in Water Colours, under the auspices of the Ladies' Association of the Hampstead Hospital, Parliament Hill, and a large sum was realised. —The secretary of the Midwives' Institute and Trained Nurses' Club wishes to thank all those who have so kindly forwarded games, &c., for our convalescent soldiers in South Africa. The contributors will be pleased to hear that several large packages were sent off last week.

### Lectures to Surgical Nurses.

By H. A. Latimer, M.D. (Dunelm), M.R.C.S. of Eng., L.S.A. of London, Consulting Surgeon, Swansea Hospital; Past President of the Swansea Medical Society; Lecturer and Examiner of the St. John Ambulance Association, &c.

### CANCER, OR MALIGNANT DISEASE.

The subject I have chosen for my lecture to-day is that of malignant diseases. At first sight you would say that this is a very extensive question, for any illness, by assuming a formidable aspect, may be said to have become malignant. But it is not in such a sense as this is that the term malignant is applied in surgery. With us it is practically synonymous with the word cancer. It bears the same relation to this fell disease as I have told you, in a former lecture, is borne by the word typhoid. Then I showed you that this word could be used as an adjective, denoting a condition of illness; that it indicated a low state of an invalid during illness; that we could speak of "the typhoid condition" of a person suffering from some ailment, as, for instance, pneumonia; and I pointed out that the word was also applied to a specific fever —typhoid fever—which is more properly called enteric fever. In an exactly similar manner we hear of a malignant attack of malarial or scarlet fever; but when a person is said to have malignant disease, then one or other of the cancers is what is meant. I say one or other of the cancers advisedly, for there are many varieties of this complaint. Pathologists by a close observation of malignant growths have classified them and divided them into many orders—the differences from an anatomical point of view being largely based upon their structure and constituent cell life. Into these refinements it is not my purpose to enter; it suffices for me to say that the great main divisions of malignant growths are into those called sarcomas and cancers. This much, however, I may tell you, as adding to the interest of my subject, that whereas the cells making up the greater part of a sarcoma are of an immature type, which exist properly in the unborn infant at the earlier period of its development, those in true cancers are of a normal character, and are really only the excessive development and growth of the natural tissues of the part they are invading. There are other distinctions which I will briefly touch upon. If a cut section of a sarcomatous tumour is examined under the microscope it will be found that the cells which make up the bulk of it are separated in all directions by blood-vessels; whilst a section of a cancer will show that the growth is permeated by fibrous bands, which make divisions of the growth. This division of the subject is not merely a scientific one, for it extends to the life history of the disease and its modes of propagation within the body; for the sarcoma makes its way into the system at large by its cells entering blood-vessels at the first seat of disease, whilst cancers infect other parts by their cells gaining access to the lymphatic or absorbent vessels, which are to be found running in the neighbourhood of the fibrous bands I have already spoken of. As to the prime cause of malignant disease generally this has not yet been determined. A great many persons are hard at work making investigations on the subject, and many theories have been advanced in this direction. Although we are yet in the dark as to its mode of origin, it is more than probable that in time this point will be definitely cleared up. In the meantime, much suggestive information is being gathered which would point to some infection from pre-existing disease elsewhere being at work. I think it is pretty well proved that the disease is apt to dog a locality, or even a certain house, and if this is the case it would go far to point to some infective agency, being the first cause. One thing I am sure of, viz., that if a part has been injured or has been affected by a localised disease, at some future time cancer is prone to develop itself there. It would seem as if a part which had at one time been so injured or diseased that some thickening of the tissues is left behind, on an apparent cure having taken place, is left with some tendency for a cancerous process to develop itself there at some later date. That this should be so we can hardly wonder at when it is considered what cancer consists of. For a microscropical examination of a true cancerous tumour shows it to be made up of a hugely-increased number of the same cells as ordinarily exist at that spot, but in a differently arranged state, and with a definite limitation. In the cancer these cells have pushed their way into adjoining parts, and have invaded the structures in their neighbourhood—they have behaved, in fact, as the name of the disease would denote, for it is called by a word which means a crab—and have fixed their claws in the surrounding tissues. This peculiar behaviour is one means by which we discover the nature of the tumour we are examining in a suspected case. Should the growth be found to have no well-defined limits, and to have incorporated itself with the structures near to it, our suspicions are aroused as to its malignant nature; and if we find enlarged absorbent glands where we know that the path of absorption from the affected part lies, we are pretty safe in our diagnosis of the disease. This local origin of cancer renders it most desirable that any swelling or tumour should be carefully examined at an early stage of its existence, and that if any suspicion exists as to its innocent or malignant nature the surgeon should act on the latter supposition. For the evidence would appear to be undeniable that there is a period in the life of every cancerous tumour when the disease is localised, and that the general infection of the system which declares itself further on is the result of absorption of infective agents through the medium of the blood vessels, or the lymphatic channels, which come from the first seat of disease. For these reasons: because a tumour, though innocent in its earlier history, may change its character and take on a malignant action; and because I believe there is a short period in which the disease is localised, I have always advocated and practised early removal. There is another justification for early removals of tumours, especially when they exist in parts which are notoriously liable to cancer, and that is, that by taking them away patients are relieved of an ever-present dreadful anxiety. The breast is known by every woman to be subject to cancer, and if a "lump," or tumour, shows itself there a dreadful anxiety seizes the patient who has it as to its nature—an anxiety which is very likely to seriously impair her health, and which may, for all we know, by lowering her vitality, in some mysterious way let in the malignant element, even if it were not present in the first instance. When the cancerous process has once started, in the majority of cases it quickly extends, and then it exhausts the bodily health by the general disturbance it excites, by the pain it causes, and by the fever which accompanies its local and extended growth.

All these factors are dominated by the variety of the primary form which has affected the sufferer; and it is a practical maxim which is the result of observation that the rapidity of progress of a case largely depends upon the looseness, or otherwise, of the cell forms in the tumour when the process has started. The matter may be put in this way: the softer and more succulent the growth is, the more rapid and malignant is its progress. Some cases of scirrhus cancer—a form which often affects the breast—in which the cells are comparatively few and the fibrous tissue has multiplied excessively, will last for years, making very slow progress either by local or general extension. In such cases there is, as a rule, much pain.

### Notes from the Camp at Maritzburg.

By an Alexandra Nurse.

NURSING THE SOLDIERS' WIVES.

November 17th, 1899.

My last communication was written before war was declared, though it was very imminent. Since then we have had lively times, and been in the midst of scares, wars, and rumours of wars. War is a reality now, and a few days ago it seemed as if we in Maritzburg were bed not knowing but that in the morning we should find ourselves surrounded by Boers. Happily, that scare has abated, as now we feel friends are here, so many troops having arrived this week. My nursing is still confined to the women and children of the regiments. Just a month ago those of the Ladysmith camp were hurriedly sent down, thus adding some 250 to my already large family of over 300. But they were welcomed, and their comfort and sickness attended to as best we could under the circumstances.

The Ladysmith contingent were quartered in the reading, recreation, and barrack rooms of the Dublin Fusiliers. As they had been exposed for hours to a heavy downpour of rain during their transit, many developed influenza, with high temperature and some enteric symptoms. In one case a mother and five children were all down at once—temperature from 103-104·6. All, however, got on nicely, with the exception of a baby six months old, whose mother, a consumptive patient, who was suffering from enteric fever, had to be removed with her to Grey's Hospital, where the baby died after a week's illness. In our own camp we have not had a single death this year, although we have had 104 cases of measles. All made good recovery. Since the beginning of March I have paid 4,509 visits, and feel gratified that I have no deaths to record.

Last week it was deemed advisable to send the women and children home. They left in detachments on November 7th, 8th, and 9th for Durban, sailing per troopship "Jelunga" on the 11th. The railway platform was a busy and most interesting sight as the women and children, with their numerous parcels and packages, were placed in the carriages. But they all left comfortably after receiving sandwiches, bananas, and bottles of milk from some kind friends. One poor woman with seven children, who had been on the sick list the previous week, got down just in time to see the train move off. She, however, did not lose her spirits over the mishap, but accepted an invitation to the house of a good Samaritan to spend the day, and at five p.m. I saw her off to join her fellow travellers, grateful and smiling. The genial Scotch stationmaster kindly gave her a first-class compartment. Two of the women who travelled from Ladysmith when the camp was cleared had to be met by the ambulance waggon and conveyed direct to Grey's Hospital, so at three a.m. I waited for them, promenading an almost deserted platform. The train duly arrived, and four corporals quickly installed them in the waggon which was ready for them. But a surprise was in store, as they brought four children not bargained for. At first I was puzzled what to do with these, but I quickly decided to take them on to Grey's and bespeak the hospitality of the kind-hearted matron, Mrs. M'Donald, till other arrangements could be made for their safe keeping during the illness of the mothers. I need hardly say that the four sleepy little mites, the eldest only five years old, received a warm welcome, and were soon in bed. Grey's Hospital has been quite a boon to us at this time, as so many of the cases, maternity and enteric, could not be nursed in open barrack rooms. A number of our wounded soldiers have also been nursed there and are loud in its praise, and I am glad to be able to state that the doctors and matron speak in very high terms of the good behaviour, gratitude, and patience of their soldier patients. They were so very anxious to save trouble and help each other all they could. The camp at Fort Napier has now been almost entirely evacuated, and the wounded transferred to the college and its grounds, now used as a hospital. The Legislative Assembly rooms have also been fitted up for the use of the wounded volunteers and caribineers. When I visited them few of the soldiers were in bed. Some were up and able to play bagatelle, billiards, &c. I suppose they were fighting their battles over again, but at a much less expense of life and limb. Altogether it has been a most exciting period, and our nerves have been kept at very high tension. My women demand much sympathy. Three of them were widowed, several knew of their husbands being wounded, and many, especially those of the 10th Mountain Battery, were in a most anxious state, as reports that it had been captured or cut down had just been received, and they could hear nothing positive before they sailed.

After they left I was advised to take a short holiday and rest ere resuming work. Of course, we still have a number of women and children, kept back on account of sickness, to care for; but these are comparatively few, so I have come to Durban for a couple of weeks. On my way down on Tuesday we passed the trains bearing the East and West Surrey Regiments to the front. They gave hearty cheers when they saw my uniform, and asked numerous questions. Passengers heartily responded with good wishes, and we gave them all we had of biscuits, bananas, sandwiches, newspapers, &c.

Next day, accompanied by two friends, I visited the Point to see the disembarkation of troops arriving in the "Hawarden Castle." We had a splendid view from the bridge of a neighbouring ship. Cheer upon cheer was given as the men mounted into the trucks and steamed away, fruit having first been liberally supplied by the crowds. A few horses were also brought on shore. At first they appeared to be very stiff and almost afraid to use their limbs, but when they were gently led up and down, they gained courage and trotted round quite briskly.

Then we made our way to the hospital ship "Spartan," and, permission being granted, we were courteously shown over by Sister Makepeace. The ship is very nicely arranged, the saloons being fitted up most comfortably for the sick and wounded, one being reserved for officers. Flowers and plants were in evidence, and gave the saloons quite a home-like appearance. An electric fan was also, I feel sure, a welcome addition. The operating theatre was a special object of admiration, but I trust that it may not be necessary to use it frequently. My experience of the sea—and I have had a good deal—is that steamers are almost always unsteady. Successful operation under such conditions must, indeed, be a triumph of skill no less than an anxiety to all concerned.

Only five soldiers were in bed, these being the more serious cases. One poor man was suffering badly from abscess in the liver. His great regret was that he had not been able to go to the front at all. On the upper deck we met friends, including wounded soldiers from our own Maritzburg camp, and a Gordon Highlander from my native city—Glasgow. These I had seen in Grey's Hospital. Now they were able to enjoy a smoke, and to tell us how and where they had been wounded. One of the Highlanders said that his friend, on gaining the top of the hill, placed his gun on a rock, and, in a hailstorm of bullets, danced the Highland fling, and then continued fighting. The men were loud in praise of their surroundings and of all that was being done for their comfort and happiness. But they were unanimous in the wish to get back as quickly as possible to help to fight their country's battles, "and just to have one more shot at the Boers."

### Help the Nurses to Help the Sick.

**Royal National Pension Fund for Nurses**, 28, Finsbury Pavement, E.C.—This Fund has during the past year maintained the uninterrupted success which has attended it ever since its establishment. The number of members who have joined the Pension Fund in 1899 shows a most satisfactory increase. Nearly £1,500 has been distributed in sick pay, a fact which speaks eloquently as to the blessing which this branch of the Fund's work must be to nurses, more especially, of course, to those working on their own account. About £3,000 was paid away in pensions and bonuses in 1899, and it is interesting to note that this amount, as compared with the previous year, shows an increase of not less than 33 per cent. The premium income—*i.e.*, payment by, or for, nurses—was close upon £70,000.

**The Junius S. Morgan Benevolent Fund** is an auxiliary to the above fund for nurses, was founded through generous contributions from nurses themselves, and raised to handsome proportions by the munificence of the Morgan family and many other friends to nurses. The work is done by volunteers, under the supervision of an influential committee, which devotes time and care to the investigation of claims and the relief of urgent cases. Hon. Secretary, Miss Rosalind Pritchard.

**East London Nursing Society.**—The object of this society is to nurse the sick poor in East London in their own homes by means of trained resident nurses, each nurse living in the parish in which she works. The extent of the society's useful work is shown by the fact that in 1898 the staff of 30 nurses attended to 5,187 persons, to whom 116,020 visits were made. Annual subscriptions and donations to the general fund are asked for. Secretary, Mr. Arthur W. Lacey, 168, Whitechapel Road, E.

**Metropolitan Nursing Association**, Bloomsbury Square, W.C.—Founded in 1875 as a Training School and Home for District Nurses who have previously gone through a full course of hospital training, and who nurse the sick poor in their own homes within a radius of a mile and a half from Bloomsbury Square. This Association is now the central training home for the Queen's Nurses. Superintendent, Miss A. H. B. Hadden.

**Queen Victoria's Jubilee Institute for Nurses.** Offices: St. Katharine's Precints, Gloucester Gate, Regent's Park, N.W.—The Institute trains nurses in district nursing, and supplies nurses to affiliated associations for the sick poor in their own homes. Applications for information should be addressed to Miss Peter, the general superintendent. Nursing associations in England, Scotland, Ireland, and Wales are affiliated with the Institute.

**"The Hospital" Convalescent Fund.**—Since the establishment of this fund many tired and delicate workers have enjoyed a much-needed change of air such as they could not possibly have secured for themselves without help. Experience has proved that it is better to let the nurses have a choice of locality rather than to send them to one settled place, and the object of the fund, namely, to provide rest for weary workers, amidst suitable surroundings, without any of that anxiety about ways and means which retards convalescence, is fully carried out. Contributions which would increase the field of usefulness are invited by the Hon. Secretaries, care of the Editor of THE HOSPITAL.

**Up-Country Nursing Association for Europeans in India.**—The chief object of this association is the provision of skilled nursing for Europeans, especially civilians, in the up-country districts of India. The association in London selects the nurses, and all expenses are paid. Hon. Secretary, Major-General J. Bonus, R.E., The Cedars, Strawberry Hill.

**The Colonial Nursing Association**, the Imperial Institute, S.W.—This valuable association was founded three years ago to supply trained nurses in the Crown Colonies and small British communities in foreign countries. Since the foundation 50 nurses have been despatched to various parts of the world, grants in aid being made where it is clearly shown to be impossible for the residents unassisted to bear entire cost of passage moneys, salaries, and maintenance. It is one that appeals to the sympathies of all, for which family is there that has not some members in distant lands, building up the Empire, and fighting with the sickness that comes with rough faring and undrained country? The Hon. Secretary, Mrs. Piggott, will be glad to receive contributions, especially as an effort is being made just now to extend its benefits to the poorer colonies.

### Nursing on the Hospital ss. "Spartan."

BY A NURSE ON BOARD.

November 14th.

AFTER a fairly good passage we arrived at Cape Town on November 4th, and outside the harbour of Durban on the 7th. It was so rough we could not cross the bar; we rolled and swung like a pendulum from 10 to 45 deg. for nearly sixty hours. Little sleep was obtained at night by anyone; in our cabin chairs, trunks, &c., raced up and down. Four times one night I tried to lash and fix them, but they soon got loose again. Meals were like a pantomime, everything seemed alive, and to lodge food safely into one's mouth was quite a feat. We were all very glad when the sea calmed, enabling us to steam into the harbour.

### A LOOK ROUND.

We went on shore in turns, and were surprised to find Durban so home-like and having such good shops. What struck us as most strange were the rickshaws, small carriages drawn by Zulu "boys," and said "boys'" head-dresses. These head-dresses represented birds or beasts, or some device—if birds, they were composed of feathers; if beasts, they had horns. These "boys" rarely run more than two years; they get bad heart disease. We found them very honest and civil.

### THE FIRST PATIENTS.

On the 11th, at eight p.m., forty-seven patients arrived—ten medical and the rest surgical cases. They were lowered into the wards in a lift, put to bed, and every man given champagne, and afterwards milk, beef tea, bread and butter, &c. They were all very tired and exhausted and glad to get to sleep. One poor man had a bullet wound on each hip, the bullet having passed through his body. Another had a bullet pass through his scalp a little above the ear and out again. Both are doing well.

### OUR VISITORS.

Crowds of visitors come daily, bringing presents suitable and unsuitable, and flowers come in not daily but hourly. All greatly appreciate the sympathy and kindness displayed, and now that we have made a list of acceptable and suitable luxuries for officers and men useful things are brought.

### THE MOVEMENTS OF THE "SPARTAN."

As soon as the next batch of sick and wounded are on board we shall take them to Cape Town; then we shall return here for more. The hospital ship "Trojan" takes our place here when we leave; she has arrived at Cape Town.

### OUR NURSES ON DUTY.

We have breakfast at 8.30 a.m., and go to our wards at 9. The surgeons go round at 10, and are at work till 12.30 in the wards. We take it in turns to be off afternoon duty, but it is not much rest unless we go on shore, as the whole time is occupied in showing visitors round. We divide the night into watches: No. I. Sister went on first night from 9 to 12; No. II. Sister from 12 to 3; No. III. Sister from 3 to 9. Second night No. II. goes on at 9, No. III. at 12, No. I. at 3, and so on in rotation.

### In a Christmas Library.

### THE CHILDREN'S SHELF.

The Little Panjandrum's Dodo. (Skeffington and Son.)

"I think you will believe in fairies before we have done with you," says one of the gnomes in Mr. Farrow's latest book, when Dick, Marjorie, and "Fidge" Verrinder were first admitted to the realm inhabited by the "wee people." They have cast their spells over us also, for we lay down the whimsical, delightful story feeling that we too have been on a trip in Fairyland, and, moreover, regret it has come to an end! Dick, who is thirteen, protests that he has had it all explained to him, and he does not believe in elves "and things of that sort" any more, but when he inconsistently praises Shakespeare's Oberon and Puck the little gnomes deride him without mercy, and will not accept his attempted justifications. By command of the Little Panjandrum the children are sent off to search for and bring back the truant Dodo. Their experiences while on this quest include a visit to the North Pole ("made in Germany"), and rides taken on the backs of the docile dolphins. They also make acquaintance with talking birds, beasts, and fishes, and all kinds of beautiful and curious things on the floor of the deep sea. Throughout their many adventures the children remain very real, human little beings, from the first moment when they patter barefoot downstairs in their night clothes to the finale, when they own to never having thought of their poor parents' anxiety during their adventurous journeyings. Mr. Alan Wright's illustrations add to the attractions of this fascinating gift book.

The Girl's Own Annual. (London: 56, Paternoster Row.)

This handsome, comprehensive volume forms a library in itself, for it is a perfect storehouse of long and short tales by various well-known and some very famous writers. A number of first-rate articles on all kinds of subjects are also among the contents, and will prove reliable sources of information as to the many lines of work now open to women and girls. All tastes are catered for, and therefore everyone will find something to interest them whether they aspire to gain profit or pleasure, or both, from the work which commends itself to each of them. The *Girl's Own Paper* is nearly twenty years old; the thousandth number has an honoured place in this season's Annual, and is pleasantly conspicuous for a fine collection of portraits of contributors.

The Courteous Knight and Other Tales. (Thomas Nelson and Sons.)

There is an attractive quaintness about the illustrations as well as the letterpress of these tales, which E. Edwardson tells us are "borrowed" from Spenser and Sir Thomas Malory. The Beast of the Thousand Tongues, which Sir Calidore, after many mishaps, succeeding in muzzling, and which he kept efficiently chained during his own lifetime, is an easily-recognised and mischievous monster. The stories of King Arthur and his knights are, of course, interesting, but even in the present moment, when war is a familiar thing, we pause amazed at Arthur's battle with the Roman Emperor Lucius, whom he killed, with Excalibur, a hundred thousand men being also slain! There is a great deal of irony in the old story of the Ape and the Fox who decide that, having the whole world before them, they only want to get that share of it which belongs to them by right. The ape craftily remarks: "I shall be content with that if I am allowed to settle myself what belongs to me by right." The after fate of the pair of rogues is equal to their deserts.

Told in the Twilight. (C. Arthur Pearson, Limited.)

This is a collection of ten of the dear old nursery stories which children love, and which maintain their popularity with successive generations of little folks. The present issue contains, amongst others, "Rip Van Winkle," "Robin Hood," and "The Pied Piper." The pages are notable for broad margins, clear type, and illustrations by Miss Blanche McManus.

Soldiers of the Queen. (Thomas Nelson and Sons.)

This is a most admirably illustrated and seasonable picture book, and the fact of its being designed and printed in Great Britain will enhance its value in the eyes of many purchasers. The spirited, well-coloured representations of the different branches of her Majesty's army are accompanied by descriptive letterpress which must prove interesting to older readers than those for whom it is primarily intended.

The same publishers have issued for quite little children a charming "Farmhouse Alphabet," and a very pretty book called "Our Pets' Pictures."

Legend-led. (The Religious Tract Society.)

Two wilful boys and their little sister Gypsy are made by Miss Amy Le Feuvre into a very amusing trio. They managed to get into about as much mischief as is possible even to lively little people whose heads are packed with legendary lore. Many of the adventures which fall to their lot are caused by their governess paying far more attention to the perusal of her own books of poetry than to the dispositions of her pupils, to whom, notwithstanding their faults, she is sincerely attached. The children have been much impressed by the history of the Quest of the Holy Grail, and Gypsy and another little girl have some disastrous experiences when they run away to look for it. The step-brother to whom Gypsy and her brothers owe their pleasant country home is regarded by them as an enemy and designated "The Ogre," before they really know him. The little girl is the first to make friends with him, and is looked upon as a kind of traitor by her brothers in consequence. A very charming Miss Helen comes at length on the scene, and as she is almost the only person in the book who really understands children, it is well for the little orphans that she agrees to marry their sorely-worried brother.

The Bond of Love.

This little book, which is published by the same society, is not like the preceding ones, for children. The heroine of Miss Margaret Thorn's story is the daughter of a hard-working London doctor and his delicate wife. When the latter goes abroad for health, Esther is sent on a visit to her relations, the Rev. James and Mrs. Barclay. The reader is introduced to these worthy people just as Mr. Barclay has been appointed superintendent of a Wesleyan circuit in Devonshire. The conversion of Esther, who has been brought up in the English Church, to Methodism, and her courtship by a local preacher, are the chief events of the book. There are, however, some pleasantly told sketches of other local characters, and in the end, having encountered a disciplinary amount of opposition, Esther is married to the man of her choice and settles down to a peaceful, if somewhat humble, future with all the zeal of a convert.

Clipped Wings.

Several young ladies share the rôle of heroine in this story, brought out by the same society as the two just noticed. The authoress (Harriet E. Colvile) manages to bring them all victoriously out of their troubles and settles them down to their duties. The piety of the worthy Mrs. Percival is so sincere that the reader can but regret the exceeding narrowness of her religious and intellectual opinions.

Heroes of the Nineteenth Century. (C. Arthur Pearson, Limited.)

The heroes of whom C. Barnett Smith gives us the histories are four in number. They are Nelson, Napier, Roberts, and Livingstone, and to each of these famous men he has devoted considerable pains and thought, with most happy results. We gain from each biography a peculiarly interesting and fresh study of those who have been active in the making of the history of the century. The present book, which is one of two uniform volumes, contains numerous illustrations besides the portraits of the heroes themselves.

The Gimcrack Jingle Alphabet. (Dean and Son.)

An alphabet in rhyme with comic pictures and in large print is certain to be popular with small children at Christmas or any other time of year.

Pictures for Little Englanders. (Dean and Son, Limited.)

Under this title Messrs. Dean have published a book which does not seem to us by any means worthy of their reputation. The pictures are highly-coloured caricatures accompanied by very up-to-date verses. It may be doubtful taste to hold up to ridicule, in a book for children, a variety of public personages, but we do not hesitate to condemn the inexcusable vulgarity of the representations of her most gracious Majesty the Queen.

The Red Rat's Daughter. (Ward, Lock, and Co.)

From the pen of Mr. Guy Boothby we may always count on getting a story full of exciting adventures, and generally, too, we can anticipate a journey by sea or land to some of the countries with which this author seems better acquainted than the majority of his readers. There is plenty of love-making in the present romance, and also many moments of intensified interest in the course of the development of a most original plot. The encounter with a Russian man-of-war must be read in its entirety, and the whole book will prove an antidote to the depressing influence of winter fogs and dull days.

Uncle Tom's Cabin. (Ward, Lock, and Co.)

A new edition of Mrs. Beecher Stowe's remarkable story contains as preface some interesting particulars of the authoress's methods of work, more particularly as regards the circumstances which led to the writing of "Uncle Tom's Cabin." No doubt some of our readers will be glad to secure a copy of this well-bound and inexpensive edition of the powerful story.

Phil and I. (Thomas Nelson and Sons.)

This admirable tale will certainly be one of the most popular gift-books of the present season, and it deserves to be a permanent favourite. The friendship of two boys of different nationalities is attended with numerous drawbacks which tend to strengthen rather than loosen the bond between them. Mr. Paul Blake has given us a good picture of school life in the end of the last century, and the "barring out" episode is capital, with its ingenious conclusion. The whole book abounds with incidents which will be heartily enjoyed by the boys and girls who make the acquaintance of "Phil and I."

Terry's Trials and Triumphs. (Thomas Nelson and Sons.)

An intelligent, attractive little Irish boy, Terry Ahearn, begins life in a wretched court known locally as "Blind Alley," in the city of Halifax. By a stroke of good luck, Terry gets a chance of a fair start in life, of which he takes advantage. He has some thrilling adventures by sea, in the time of the American Civil War, and his whole history is such as is certain to recommend itself to all young folks who love a well-told and stirring story. Mr. Macdonald Oxley's name is associated with other deservedly popular story books.

Signors of the Night. (C. Arthur Pearson, Limited.)

Few writers know Venice as Max Pemberton does, and fewer still can make the daily life of the fair city an actual reality to his readers. The events chronicled in the volume before us are all connected with Fra Giovanni, the soldier-monk of Venice, and they form a series of exciting, powerfully-told episodes. We can cordially recommend this book to the attention of those who want a pleasant change of thought, and we feel sure that from the first page to the last they will find themselves in a truly Venetian atmosphere. The illustrations are excellent.

Two Years Ago. (Ward, Lock, and Co., Limited.)

This latest edition of an old favourite comes forth in a pretty red binding, and contains illustrations. Turning over the pages in the lingering fashion in which we handle the books of Charles Kingsley and other standard writers, we come to the description of a scene in the Strand. Dr. Thomas Thurnall, one of Mr. Kingsley's manly heroes, is starting for the Crimea next day. "He walked out into the Strand . . . to smoke in the fresh air . . . passed one of the theatre doors; there was a group outside, more noisy and more earnest than such groups are wont to be; and ere he could pass through them a shout from within rattled the doors with its mighty pulse, and seemed to shake the very walls. Another, and another! What was it? Fire? No; it was the news of Alma. . . . And the group surged to and fro outside, and questioned and rejoiced. . . . They questioned Tom, taking him for an officer, as to whether there were many killed. . . . 'I am no officer; but I have been in many a battle, and have seen how they fight; there is many a brave man killed, and many a one more will be.' 'Oh, does it hurt them much?' asked one poor thing. 'Not often,' quoth Tom. 'Thank God! Thank God!'"

### To Nurses.

**In order to increase and vary the interest in the *Mirror*, we invite contributions from any of our readers in the form of either an article, a paragraph, or information, and will pay a minimum of 5s. for each contribution. All rejected manuscripts are returned in due course, and all payments for manuscripts used are made at the beginning of each quarter, i.e., January 1st, April 1st, July 1st, and October 1st.**

### Echoes from the Outside World.

### AN OPEN LETTER TO A HOSPITAL NURSE.

The news from South Africa this week so far has been very trying to one's patience. We are sure that all will come right in the end; but the tidings of Lord Methuen's check at Modder River, with heavy loss, including the death of General Wauchope, who was killed in action, following the serious reverse of General Gatacre at Stormberg, must profoundly impress everyone who was not previously impressed with the extremely arduous nature of the task which lies before the British troops, and the perils which they must still encounter. There is never a time when a reverse is welcome, but at the moment when the little force near Stormberg met with their disaster it seemed as if a successful engagement would have done so much to turn the fortunes of the war in the direction we desire. The Free Staters had begun to grow dissatisfied with the opposition from our troops, and were known to contemplate returning home after one more stand. Now they will take heart of grace with the arrival at Bloemfontein of 600 British prisoners, and begin once more to believe the assertion of the Boers that victory is certain to ensue in the long run. Also the disaffected Dutch in Cape Colony, who have the last few weeks been so undecided whether to join the enemy or to keep quiet, will, it is feared, see good reason to unite themselves to the enemy. But none of these considerations at all justify the harsh and hasty judgment upon General Gatacre which has been passed not only by private individuals but by some of the newspapers. It is so easy to sit in an armchair at home and criticise the commander in the field. Any simpleton can do that, but it takes a wiser woman to hold her tongue. Lord Durham's statement, made on hearsay evidence, that General Gatacre overworked his men in the Soudan, was both unwise and uncharitable, even if it were true, and a peer who was once a lieutenant in the Coldstream Guards can scarcely be regarded in the light of a capable soldier, whose opinion should carry weight. Ladysmith seems not only holding its own, but General White and his men are making successful sorties on their own account. Kimberley is still safe, but poor little Mafeking is hard pressed, as the bombardment is producing more effect, and the rations are being reduced.

---

Letters from Natal this week throw a welcome light upon the incident of the "Sumatra," and explain why the wounded soldiers were allowed to lie waiting on the wharf at Durban for two hours, and were then only given the most unappetising food, quite unfitted for invalids. The military doctor at Maritzburg sent the poor fellows down too soon, before they were expected, so that it is easy to understand the Durban commandant did not know what to do with them, for though he felt he was bound to find them shelter, he was conscious that no comforts had as yet been provided for them, nor adequate arrangements made. Now my correspondent says that everything that can be done to alleviate pain and suffering is being done, and that there is no need for the inhabitants of Durban—who have already spent large sums in helping the refugees and those whose husbands have volunteered for the front—to do more than visit and cheer the invalids, and take them flowers or dainties. The "Spartan" and "Trojan," both hospital ships, have arrived, and are pronounced "perfect in every detail, and the nurses charming." When the army sisters came they expressed surprise that there were no letters for them with instructions from the sisters at Ladysmith, and were quite unprepared for the announcement that the town was cut off from all communication with the outside world. The enthusiasm when the soldiers land is intense; even the children buy cakes and sweets with their own money and give them away, and baskets of fruit of all kinds are always forthcoming from the elders. The gratitude which the soldiers show seems to impress the Durban folks vastly, and some of the naval men off the "Terrible" who were quartered in one lady's garden subscribed amongst themselves before they left, unprompted by any suggestion of their officers, went down into the town to buy a little silver manicure case, and presented it with words of thanks to their hostess. You may have thought it strange that although my friend in Natal spoke of the wounded whom she saw on board the "Sumatra," when the vessel arrived in the Albert Docks, it was ascertained that none of the soldiers had ever been in action. The reason of the change was that the "Sumatra" only took the wounded as far as Capetown, and left them at the Wynberg Hospital, the vessel proceeding to London with invalid soldiers who had fallen ill in their country's service, but had not been in action.

---

When there is so much in the papers to sadden, it is pleasant to read that it is not only on the battlefield our troops can show their bravery. An account just to hand says that when the transport "Ismore," on its way to the Cape, was wrecked, during the critical time when everyone expected the vessel to go down, the conduct of the Hussars was most praiseworthy. Not a man but stuck to his post, and everyone was as cool as if he had been on parade. Another interesting item is that the coloured lepers on Robben Island have opened a fund in aid of the sick and wounded Imperial troops in South Africa, and have forwarded their first donation to Sir Alfred Milner. Such an offering from those whose illness debars them from active life themselves is very touching.

---

The Earl's Court Exhibition next year is to be devoted entirely to Woman, her art and her work. As such it will be especially interesting to all of us, and nurses and their labours are sure to take a prominent part. So, too, will the agricultural woman—a product of the end of the century. Of course, for years farmers' wives and farmers' daughters have made butter and cheese, and thus helped the family exchequer, but it is only lately that young ladies of good education have seen that there is an opening for them in the capacity of growers of flowers and fruit, rearers of poultry, and keepers of bees, from a business point of view, and as a means of making themselves self-supporting. Lady Warwick's Hostel in the most pleasant suburb of Reading has just celebrated its Founder's Day, and at the meeting on Saturday, it was frankly admitted by Mr. Asquith that at the outset he had had strong doubts of the ultimate success of the scheme for putting cultured women on the land, but he had been converted by results. These results I have seen for myself. In twelve months the number of inmates has risen from twelve to nearly forty, and this term a second "hall of residence," called Maynard Hostel, has been opened. It is close to the original establishment, and is fitted up in the same admirable manner. Attached to each house is a considerable quantity of land, and every student has her own plot of ground to cultivate. On the occasion of my visit I saw several young ladies busily occupied in tending poultry runs and glass houses, whilst some were hard at work digging, and all looked the very picture of health. There is no doubt that the movement is a valuable and popular adjunct to Reading College.

### Christmas Shopping.

### A FEW SUGGESTIONS.

Already the streets of London are thronged with a feminine crowd all occupied in collecting the store of treasures to be dispersed at Christmas day. To watch the countenances one

A Pretty Chatelaine.

would imagine the cares and worries of life overwhelmed the thoughts of the would-be purchasers. And truly, although their sole object is to add to the pleasures of a festive season, the shops are full of anxious and weary ladies suffering from

A Useful Ink Bottle.

an *embarras de richesse* in novelties and pretty trifles around them, and a poverty of ideas on their own part. Any suggestions are valuable in such times of doubt. One idea may lead to another, and so a successful purchase be made. Now supposing our readers are in search of a souvenir for a friend that will be useful, pretty, and not too ephemeral, we would suggest that they should pay a visit to Messrs. Heming's establishment—the well-known jeweller and silversmith at 28, Conduit Street. They have all sorts of gifts appropriate for male friends, pencils, cardcases, match-boxes, whist markers, cigarette cases, cigar cutters, and a host of other attractive novelties at quite surprisingly low prices. Our illustration shows a handsome yet simple ink pot, useful to either sex, but more especially useful for a ward sister when accompanying the physician on his rounds. The lovely chatelaine will appeal to all nurses, from the matron to the probationer. The workmanship is excellent, and the whole effect most elegant. For combined presentation we would suggest a delightful little solid silver clock, only £3 17s. 6d., or for a little larger expenditure one of those fascinating clocks in wood or leather case supplied with electric light for night use. There is, too, quite the prettiest little silver tea set at the most moderate price we have yet seen one. Indeed, it would be difficult not to find a suitable gift for the most fastidious at this establishment. Next we would remind our readers that no more welcome present can be found than a bottle of good Eau de Cologne. We all know that the 4,711 Eau de Cologne—the depot for which is at Mühlen's, 62, New Bond Street—is quite the best to be had. Here, too, are to be found the celebrated Rhine violet, Maréchal Niel and other perfumes, besides the best of soaps and toilet accessories of all sorts. When you are in Bond Street go a little further, to Messrs. Harris and Sons, at 25, Old Bond Street, on the first floor. Here you will find a charming exhibition of art embroidery and appliqué work on linen. This is adapted to the decoration of all kinds of useful articles. The invalid's tray is one of the daintiest novelties we have seen this Chirstmas. The teacup and plate or little soup basin are served upon embroidered linen cloths, with serviette to match. Any colour and design can be chosen. The souvenir for absent friends is a little folding photo frame for the pocket—midget size—worked in forget-me-nots. This can easily be enclosed in a letter. Sachets, writing cases, tea cosies, and numbers of other useful and pretty articles are displayed to tempt the purchaser, who will certainly find something unique to carry away. In poor homes, Chivers' convenient and excellent custard powder and jellies will form welcome dainties not taxing the culinary skill. The flannelette blankets are even better and cheaper this year than ever, and now that wool blankets are higher in price they will be still more popular. Christmas cards are everywhere, and very pretty they are. But it is difficult to find anything very new in design. Messrs. Speight, photographers, at 178, Regent Street, are issuing very pretty photograph cards with Christmas greetings, and their Baby s Album, to record events in baby's life, would please the heart of any mother.

Messrs. Garrould are showing all kinds of novelties at their establishment in Edgware Road. Nothing will be easier than to pay a visit to their bazaar for the purpose of equipping a Christmas tree. There everything is at hand desirable for the purpose. Toys and amusements for children of all ages, pretty gifts for the elders. The cheapness of the dolls is quite astonishing. Fancy a good-sized wax doll, with really pleasing feature and curling hair, for only 6¾d.; whilst jointed dolls, dolls with eyes that move, not merely close; dolls that laugh, cry, and sleep at the will of the owner, and with a host of other desirable attributes, are everywhere. Mechanical toys, Santa Claus stockings, sweatmeats surround one. And not only need one confine oneself to pleasing the little ones. Articles to satisfy maturer taste are well provided for also. We noticed especially the "Red Cross Watch," with second dial, for 8s. 6d. only, and excellent ebonised brushes with silver initials for 1s. 11½d.—quite a ridiculous price. There were mirrors and all articles for the toilet table equally cheap and attractive. We noticed also a very good selection of purses and other useful things, which show that Messrs. Garrould have successfully studied the wants of the Christmas shopper.

### Everybody's Opinion.

[Correspondence on all subjects is invited, but we cannot in any way be responsible for the opinions expressed by our correspondents. No communication can be entertained if the name and address of the correspondent is not given, as a guarantee of good faith but not necessarily for publication, or unless one side of the paper only is written on.]

### BABY'S CORD.

"Perplexed" writes: I should like the nurses who so promptly came to my assistance to know the result of my different treatment of the cord. It was not from the first an ideal one; the skin of the abdomen reached almost an inch up the cord, and where the two meet seemed a little inflamed, as if the cord had been dragged on during labour and caused slight hæmorrhage. However, I did my best, and was rewarded by being able to leave a perfectly healed and flat navel. I cut the linen (old handkerchiefs which I had previously washed and dried) according to the instructions given by "For Baby's Sake" and "Anxious," I used the starch, zinc, and boracic powder, and used it freely. I changed the dressing daily, and found no difficulty in removing it, although I did not immerse the infant until the cord dropped, which happened on the sixth day, leaving the navel a little moist; but by continuing the dressing it healed in less than a week after. I must again thank "G. A. B." for so kindly offering to send me the cut linen and fuller explanations, but all had written so clearly that I did not think it right to trouble her. I did not see "Nurse Agnes's" letter until half through the case, but I like her idea of placing a pad under the cord.

### AN EASY SWINDLE.

"An Unfortunate Nurse" writes: Would you be kind enough to expose a swindle that I have been made the victim of? On Saturday last a lady called on me in the North of London representing that she had been sent by a neighbouring chemist to whom I am known. After a lengthy conversation she engaged me to nurse her mother, whom, she said, was suffering from bronchitis. The engagement was for three or four months, as the patient was ordered south for the winter. I was to go by the 9.40 train on Monday morning to Watford, and the carriage would be waiting. The address given me was "Mrs. A. Trevelyan, Rosedene, County Avenue, Watford." After this had been arranged my visitor apologised that she could not give me my travelling expenses as she came away without her purse, and did not discover it until she got in the 'bus owing to having a pass on the line. She then looked out a train back to Watford, and said she could not get back from there to London again before a certain shop was closed; and—well, to make a long story short—she borrowed £1 from me, promising to post it off from Watford on Saturday night so that I received it early on Monday morning, and also expenses. I need hardly tell you that I did not receive a letter. But I went to Watford, to find that there was no one to meet me, neither was the name or place known. Then I went to the police office, and learned that there is no such place as County Avenue in Watford. The police were kind enough to telephone to the next station to make inquiries, with the same result. The woman also mentioned a Nurse Macdonald whom she came to engage, and who is out of London. My money is, of course, lost to me. I am merely writing to save, I hope, other hard-working nurses from being victimised in a like manner. It is a serious loss to me, as I have children depending on my earnings as well as myself.

### THE APPEAL OF THE KILBURN SISTERS.

"E. S. B." writes: In reference to your Note headed "The Appeal of the Kilburn Sisters," may I ask if the writer knows anything about this noble band of women or the work they carry on? As to nursing qualifications, personally I know that one, who is, I believe, mentioned as having already sailed to organise the work of relief in South Africa, was trained at the London Hospital, and held appointments as sister and matron in various other hospitals before joining the Kilburn Sisterhood, and since then has done much nursing among our sick poor. Surely such a one is as qualified for the work of nursing as those appointed by the War Office; and who shall say, after reading the lists of wounded, that the 56 nurses already in South Africa are more than enough for the work to be done in alleviating the sufferings of our men? May God bless and help the Kilburn Sisters, as well as the other brave nurses who have gone to do their duty at the front.

### COTTAGE HOSPITAL MANAGEMENT.

"I. M." writes: I was much interested in the letter by the "Hon. Member of Committee" in respect to cottage hospital management. In this town there is a hospital of ten beds for medical and surgical cases, and six for infectious cases; the whole is under a trained nurse as matron. She has one probationer and one servant. As a rule there are six or seven beds occupied all the year round; in the infectious block there are often several cases. One nurse is expected to nurse the whole of the patients, and very often there are several surgical cases as well. A laundress is allowed once a fortnight. The matron has the entire nursing charge of the whole place, and does the cooking and housekeeping also. The late matron was here for nearly four years, and during the whole time she had only one month's holiday. In the summer her health broke down through overwork and worry. She was at once ordered to resign. She worked very hard for four years; I think she was entitled to a little more consideration from the committee and medical men of the town. But as far as I can see, the committee are not used to trained nurses, and therefore do not know how to treat them when they have them. For the four years the number of cases treated in the hospital was from ninety to one hundred, and some of them were very severe, requiring night as well as day work. The matron was expected to do both, in addition to all the other duties. The matron has no voice in the choosing of the probationer; she is shown by the medical committee. One of the three members of the medical staff often does not come near the hospital for several days at a time, and of course this adds to the matron's work. I could write a book on the subject, but perhaps I have said enough at present.

### THE BATHING OF MALE PAUPERS, AND OTHER QUESTIONS.

"Disgusted" writes: The two cases mentioned by "Inquirer" will bear no comparison. She asks why should the words of Scripture, "to the pure all things are pure," be so misquoted? Every trained nurse and woman with common-sense knows that her duty as regards male patients bathing is to prepare the bath and keep within call. If he is unable to bathe himself the only alternative is a "blanket bath," performed, of course, by the nurse herself as her duty and pleasure, for "to the pure all things are pure," and purity is pleasure. It is not stated that the nurse who lost her post rather than overstep her duty, refused to blanket bath the "male pauper," and every right-minded woman and nurse commends her for her noble womanly conduct. Now for the "lady nurse" who can "catheter." It is only a comparatively few nurses who are able to learn this, and one who has done so has every right to use it in her advertisement. Such an advertisement would be useless for an artisan, as if he required such attention he would be most probably in a hospital or infirmary, where the medical officer would perform this office for him. But there are many more or less chronic cases ("gentlemen") to whom such a nurse is invaluable, and it is to meet the need of such as these that the "lady nurse" advertised. The "difference," "Inquirer" finds the seeing of which so great a difficulty, is that it may be necessary for a nurse "to catheter" if she would be a true help to her patient; and having learnt, I am quite sure that every true nurse would use her skill for the benefit of any patient, be he pauper or gentleman, should the need arise. But it is never necessary for a nurse to sacrifice her womanly modesty, and to outrage every law of decency.

### OUR CLOTHING DISTRIBUTION.

Some further welcome contributions have come in during the week for our clothing distribution. From far-off Natal Mrs. Roberson kindly sends us a box containing thirteen garments. The home helpers are E. V. (two), Nurse Willson (nine), E. M. Williams (two), Policy 3,920 (five), and Policy 1,081 (six).

### Appointments.

SWANSEA HOSPITAL.—Miss Margaret Bridger has been appointed Matron. She was trained at the General Infirmary, Huddersfield. She has since been staff nurse and in charge of the operating theatre at the same institution; head nurse at the Derbyshire Hospital for Women; sister of female medical and gynæcological wards at the Wolverhampton and Staffordshire General Hospital; and night superintendent from October 3rd, 1898, to November 11th, 1899, at the Swansea Hospital.

INFECTIOUS HOSPITAL, KINGSTHORPE, NORTHAMPTON.—Miss Laura Annie Upton has been appointed Nurse-Matron. She was trained at the Monsall Fever Hospital, and Royal Infirmary, Manchester. Subsequently, she has held the posts of sister at the Hull Sanatorium, sister at the Plaistow Fever Hospital, assistant matron at the Hornsey Isolation Hospital, and ward sister at the Borough Hospital, Wolverhampton.

GRANTHAM HOSPITAL.—Miss Jeanie Chapman, the new matron, wishes to make corrections in the details of her appointment, which were inserted as supplied to us last week. Miss Chapman was trained at the Western Infirmary, Glasgow, for three years, and had her fever training at the South-Eastern Hospital, New Cross, London.

GUERNSEY FEVER HOSPITAL.—Miss Margaret Banks has been appointed Nurse-Matron. She was trained in the City of Glasgow Fever Hospital, Belvidere, and has since been charge nurse for seven years in the Branch Hospital, Kennedy Street, Glasgow.

### Minor Appointments.

SWANSEA HOSPITAL.—Miss Clara Shelbourne has been appointed Sister of Female and Children's Wards. She was trained at the Fever Hospital, Bagthorpe, and at Swansea Hospital. She has since been nurse and staff nurse at the latter institution.—Miss Bessie Butler has been appointed Night Superintendent. She was trained at the General Hospital, Bristol, where she has since been nurse, sister of out-patient department, night superintendent locum, and sister of female medical wards.

ASTON UNION WORKHOUSE INFIRMARY, GRAVELLY HILL.—Miss Fanny Brotherton has been appointed Head Night Nurse. She was trained at Toxteth Township Infirmary, and has since been nurse in the same institution, nurse in the Workhouse Infirmary at Chelsea, and nurse in charge of infectious cases at Prescot Infirmary, Whiston.

WAKEFIELD UNION INFIRMARY.—Miss Bertha Snape has been appointed Night Sister. She was trained at Birmingham Workhouse Infirmary, and holds the L.O.S. certificate. She has been sister of the Wakefield Union Infirmary since last March.

BURY UNION WORKHOUSE.—Miss M. E. Ball, the newly-appointed superintendent nurse, wishes to state that she possesses the L.O.S. certificate.

### Where to Go.

DOWDESWELL GALLERIES.—An exhibition of Mr. Mortimer Mempes' most recent Dry-Points is open at these galleries, 160, New Bond Street, this week.

### OUR CONVALESCENT FUND.

M. R. has very kindly sent us 7s. for this useful fund.

### Notes and Queries.

**The contents of the Editor's Letter-box have now reached such unwieldy proportions that it has become necessary to establish a hard and fast rule regarding Answers to Correspondents. In future, all questions requiring replies will continue to be answered in this column without any fee. If an answer is required by letter, a fee of half-a-crown must be enclosed with the note containing the enquiry. We are always pleased to help our numerous correspondents to the fullest extent, and we can trust them to sympathise in the overwhelming amount of writing which makes the new rules a necessity.**

**Every communication must be accompanied by the writer's name and address, otherwise it will receive no attention.**

*South Africa.*

(117) I shall esteem it a great favour if you will kindly tell me whether any volunteer nurses' services will be acceptable to any of the nursing staffs now going to South Africa. Several ladies and myself would be glad to help in any capacity if there is an opening. Can you tell me of any hospital where we could get even a little training? We are capable, energetic women, and would give our services gladly if we can be of any use.—*Ethel Beal.*

Only the services of exceptionally qualified trained nurses are accepted for South Africa. There are plenty of hospitals which are glad of suitable probationers. See "The Nursing Profession: How and Where to Train" (2s., from the Scientific Press).

*Ionda.*

(118) I write respectfully to ask if any nurse can give me the address where I can get a hair tonic called "Ionda?" None of the chemists here can tell me where to write for it.—*M. F. B.*

Can any reader help our querist?

*R.B.N.A.*

(119) Will you kindly let me know how I can become a member of the Royal British Nurses' Association?—*S. R. L.*

Write for particulars to the Secretary, 17, Old Cavendish Street, W.C.

*Home Remedies.*

(120) A friend of mine, mother of a family, has asked me if there is any book published for lay readers giving the quantities, &c., of simple drugs and remedies which any head of a household may be called on to administer, e.g., the proper dose for child and adult of spirits of camphor or spirits of nitre for a cold, the proportion of mustard to linseed in a mixed poultice, and so on? The bottles even of stock remedies of this kind are not always labelled, and a book of directions, in simple language and strictly to be relied on, might often be of great use. I shall be grateful if you can tell me of any.—*A Nurse.*

"A Handbook for Nurses," by J. K. Watson, M.D. (Scientific Press, 5s.), would probably give a mother all the information she requires, or "Chevasse's Advice to a Mother on the Management of Her Children." (J. and A. Churchill, 2s. 6d.)

*Training School.*

(121) Will you kindly let me know if the Sheffield Union Infirmary is considered a good training school, and if its certificate would qualify the holder for a good appointment afterwards?—*I. D. M.*

As Sheffield Workhouse Infirmary has a resident medical officer and 400 beds the training would be recognised by the Local Government Board as qualifying the holder of its three year certificate for the post of superintendent nurse.

*Mask Faces.*

(122) Will you kindly inform me where I may procure artificial or mask faces?—*M. B.*

If "M. B." refers to masks such as are sometimes used by surgeons when operating on the throat she should apply to a surgical instrument maker.

*Certificates.*

(123) Would you kindly tell me if a paying probationer's certificate for one year is of the same value as that of a non-paying probationer's for two years, both being granted by same hospital? 2. Also if there is any demand by associations for qualified midwives?—*Nurse Margaret.*

A two-year certificate is always of more value than that for one year. Every nurse, however, ought to make a point of holding a three year certificate. 2. There are sometimes advertisements by associations for maternity nurses. See our advertisement columns, or advertise yourself.

*To Disinfect.*

(124) A lady superintendent would be glad to hear of any sanatorium or home, within 30 miles of Guildford, where a nurse could be received after infectious cases before resuming her duties on private staff?

The Nurses' Hostel, Francis Street, N.W., offers facilities for nurses to disinfect.

*Cottage Hospital.*

(125) Would you kindly tell me if a nurse who holds a three years' certificate from a cottage hospital of 40 beds is eligible for training in a military hospital? Also whom must I apply to at the military hospital? —*Shamrock.*

A three-year certificate from an institution which does not maintain a resident medical man is not of much value apart from private work. For acceptance by the military hospital candidates must have had three years' training in a general hospital. Applications for full particulars should be sent to the Secretary, Army Nursing Service, 18, Victoria Street, Westminster.

*Maternity.*

(126) Being a young married woman and nearing my confinement, I wish to ask you if there are any lying-in charities in Birmingham that I could enter? I am now in apartments, where my illness would not be convenient.—*E. H.*

The address of the Birmingham Lying-in Charity is 71, Newhall Street. Apply to the secretary.

## Figures and Tables

**Figure f1:**
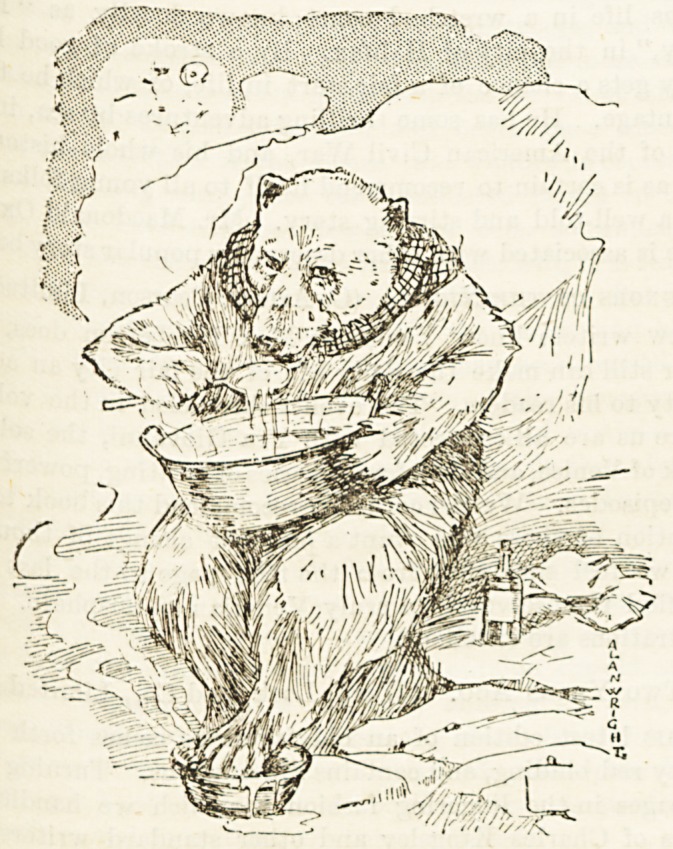


**Figure f2:**
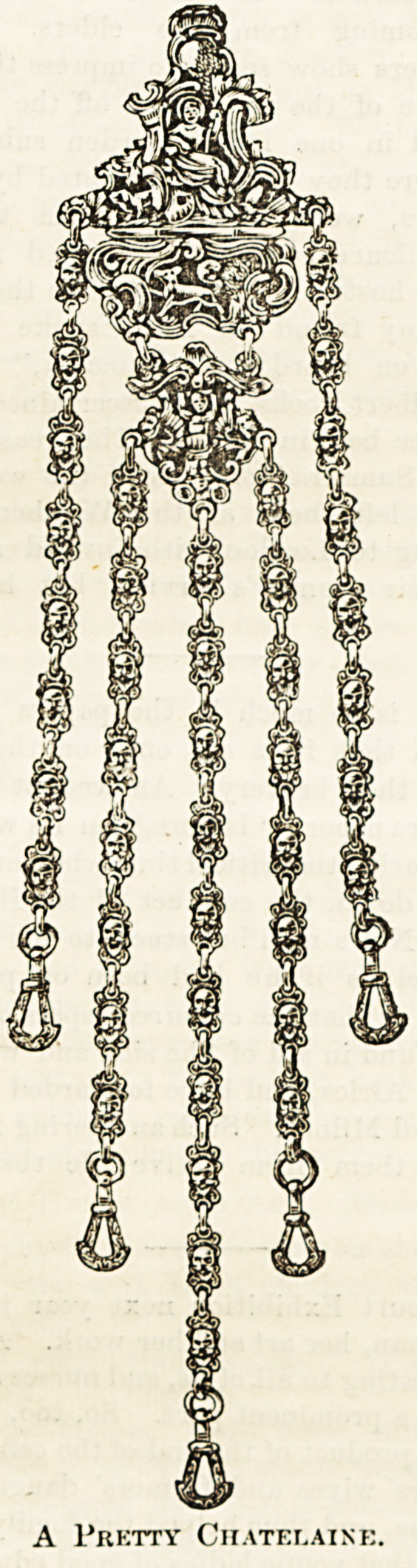


**Figure f3:**